# Reproducibility of African giant pouched rats detecting *Mycobacterium tuberculosis*

**DOI:** 10.1186/s12879-017-2347-3

**Published:** 2017-04-24

**Authors:** Haylee Ellis, Christiaan Mulder, Emilio Valverde, Alan Poling, Timothy Edwards

**Affiliations:** 1 Anti-Persoonsmijnen Ontmijnende Product Ontwikkeling – APOPO, Sokoine University of Agriculture, Morogoro, Tanzania; 2 Waikato University, Hamilton, New Zealand; 3 Amsterdam Institute for Global Health and Development, Amsterdam, The Netherlands; 4 Vanderbilt University Medical School, Nashville, USA; 5 Department of Psychology, Western Michigan University, Kalamazoo, MI 49008-5200 USA

**Keywords:** Tuberculosis, Diagnosis, Reproducibility, Detection rats, Inter-rater reliability, Intra-rater reliability

## Abstract

**Background:**

African pouched rats sniffing sputum samples provided by local clinics have significantly increased tuberculosis case findings in Tanzania and Mozambique. The objective of this study was to determine the reproducibility of rat results.

**Methods:**

Over an 18-month period 11,869 samples were examined by the rats. Intra-rater reliability was assessed through Yule’s Q. Inter-rater reliability was assessed with Krippendorff’s alpha.

**Results:**

Intra-rater reliability was high, with a mean Yule’s Q of 0.9. Inter-rater agreement was fair, with Krippendorf’s alpha ranging from 0.15 to 0.45. Both Intra- and Inter-rater reliability was independent of the sex of the animals, but they were positively correlated with age. Both intra- and inter-rater agreement was lowest for samples designated as smear-negative by the clinics.

**Conclusion:**

Overall, the reproducibility of tuberculosis detection rat results was fair and diagnostic results were therefore independent of the rats used.

**Electronic supplementary material:**

The online version of this article (doi:10.1186/s12879-017-2347-3) contains supplementary material, which is available to authorized users.

## Background

African giant pouched rats (*Cricetomys ansorgei,* previously *Cricetomys gambianus;* see [1]) have been used in Tanzania since 2007 to detect tuberculosis (TB) by sniffing sputum samples previously evaluated by light microscopy, and in 2013 operations were extended to Mozambique. Such second-line screening increased the new-case detection rate for presumptive TB patients from Morogoro and Dar es Salaam, Tanzania by 44% in 2009 [2] by 43% in 2010 [3], and by 39% in 2014 (Poling et al., under review). In 2014, it also increased the new-case detection rate in presumptive TB patients from Maputo, Mozambique by 53% (Poling et al., under review).

These results suggest that pouched rats can be of practical clinical value in regions where light microscopy is the standard TB diagnostic. The animals respond to the volatile compounds produced by tuberculosis bacterium, but not to volatiles produced by similar mycobacteria, or to volatiles that occur in the presence of both TB and other microorganisms [4]. A trained rat can evaluate as many samples in 20 min as a lab technician using conventional light microscopy can do in four days [5], and the rats are more sensitive, although their specificity is somewhat lower [6, 7]. Pouched rats can live for up to eight years and have simple care and husbandry requirements [8]; both characteristics increase their potential value as TB-detecting animals.

If, however, the rats are to be of general value, they must perform reliably. A major objection to using animals for operational disease detection is that behavior is variable and it cannot automatically be assumed that different animals will react in the same fashion to the same scent, or that the same animal will react in the same way to that scent on different occasions [9]. Although the accuracy of individual rats and rats as a group relative to culturing has been reported [6, 9], which is arguably the best indication of their clinical value, data regarding their intra-rater and inter-rater reliability in evaluating sputum samples would also be a helpful measure. The objective of the present study is to determine the reproducibility of rat results. Because prior studies have demonstrated that the age [10, 11] and sex [12, 13] of animals sometimes affect their performance in odor-detection tasks, reproducibility was evaluated as a function of these variables.

## Methods

### Subjects

Twenty-two rats working in two operational groups during the 18-month study period were included in the study. Both groups consisted of 11 rats, with a median age of 3.8 years (IQR = 3.7) in Group 1 and 2.4 years (IQR = 1.8) in Group 2. There were three females in Group 1, and six in Group 2.

### Samples

Two sputum samples were collected from presumptive TB patients who visited directly observed treatment, short course (DOTS) centers between March 1, 2014 and August 31, 2015. Patient ages ranged from 1 to 87, and 54% were male. Each sample was evaluated at the DOTS center where it was provided, by light microscopy after Ziehl-Neelsen staining, and then shipped to APOPO’s lab (Anti-Persoonsmijnen Ontmijnende Product Ontwikkeling) for evaluation by the detection rats. Samples that were classified as TB-positive by DOTS centers were used to arrange reinforcement for correct identification of positive samples by rats during evaluation sessions. Sessions were planned so that DOTS-positive samples constituted approximately 10%, with low-bacilli count samples prioritized over 1+, 2+ and 3+ samples. A total of 11,869 samples, 1704 DOTS-positive and 10,165 DOTS-negative were evaluated.

### Procedure

Details regarding the training and maintenance of TB-detection rats are provided elsewhere [2, 6, 7]. In brief, evaluation sessions took place in two rectilinear cages. Sputum samples were placed in 10 pots located immediately below holes in the cage floor and a rat indicated the presence of a TB-positive sample by pausing for at least 3 s with its nose in a particular hole. Human observers recorded such indicator responses for rats in Group 1. Indicator responses were recorded automatically for rats in Group 2 when the rat’s nose broke a photo-beam situated inside the hole. Breaking this beam triggered pellet delivery from a pellet dispenser magazine (ENV-203-94, MedAssociates, Georgia, VT) via custom designed software (MS visual basic). When appropriate, rats in Group 1 received food reinforcers (rewards) in the form of a mouthful of smashed banana and avocado delivered manually via a syringe inserted through a hole in one end wall. For rats in Group 2, food reinforcers were banana-flavored pellets (OmniTreat 5TCY™) automatically delivered via a port in the cage wall.

The rats used in the present study initially received food for exhibiting successive approximations of the indicator response, placing the nose in a hole above a TB-positive sample for several seconds. In standard operations, a rat received food when it paused above a DOTS-positive sample, but at no other time. Two groups of 11 rats evaluated a separate set of up to 100 samples twice each day. Therefore, each sample was evaluated 22 times. Any sample classified as DOTS-negative, but indicated by at least one rat, was further evaluated by fluorescence microscopy of concentrated sputum and any patient who provided a sample found to be TB-positive was reported to the appropriate DOTS center for follow-up and treatment [14]. Each rat’s evaluation of every sample (i.e., whether or not it emitted an indicator response) on each presentation was recorded and these data were used to calculate the rats’ reproducibility. Quality control checks were conducted at least once a week to ensure accurate data recording and adherence to session protocols.

### Statistical analysis

#### Intra-rater reliability

Yule’s Q was used to estimate intra-rater reliability, i.e., the degree to which a rat is consistent with itself. It is a linear transformation of the odds ratio [15] and is calculated by:

$$ Yul{e}^{\prime } s\  Q=\frac{(AD)-(BC)}{(AD)+(BC)} $$

where A is the number of samples that were indicated both times they were presented, B is the number of samples indicated on the first presentation only, C is the number of samples indicated on the second presentation only, and D is the number of samples that were not indicated on either presentation. Q can range from −1 to 1, with positive values indicating frequencies of agreements greater than expected by chance and negative values indicating frequencies lower than expected by chance. Q values provide an indication of agreement; thresholds for small (.20), moderate (.43), and large (.6) effects are based on those given by the odds ratio [16].

Differences in Yule’s Q scores between DOTS-positive and DOTS-negative samples were tested using the sign test, the correlation between Yule’s Q and age was calculated using Spearman’s correlation coefficient, and differences in Yule’s Q between male and female animals were evaluated with the Mann-Whitney U test.

#### Inter-rater reliability

Krippendorff’s alpha was used to measure inter-rater reliability, i.e., the degree to which multiple raters agree with each other. Alpha can be applied to any number of observers and categories, measured in any metric, and do not require a minimum sample size [17]. It is calculated by:

$$ \propto =1-\frac{D_o}{D_e} $$

where D_o_ is the observed agreement among values assigned to units of analysis:

$$ {D}_{o\kern0.5em }=\frac{1}{n}\ \sum_c\sum_k{o}_{c k\  metric}{\delta}_{c k}^2 $$

and D_e_ is the disagreement one would expect when the coding of units is attributable to chance rather than to the properties of these units:

$$ {D}_e = \frac{1}{n\left( n-1\right)\ }\sum_c\sum_k{n}_c\bullet {n}_{k\  metric}{\delta}_{c k}^2 $$

*o*_*ck*_ *, n*_*c*_ *, n*_*k*_ *and n*, refer to the frequencies of values in coincidence matrices; when all observers perfectly agree, D_o_ = 0 and α = 1, i.e. perfect reliability. When agreement is no more than chance, D_o_ = D_e_ and α = 0 (1). An α of .3 to .5 is considered fair agreement [18]. As we assumed high intra-rater agreement, inter-rater reliability was calculated using results from the first evaluation of each sample by all rats in each group. The two groups did not evaluate the same samples, and were therefore analyzed separately. Groups one and two evaluated 5105 and 6764 samples, respectively.

*The* sensitivity, true positives (TP), and false negatives (FN) were calculated for detecting Ziehl-Neelsen sputum smear microscopy positive TB patients as conducted in the DOTS centers. The specificity and positive and negative predictive values were not calculated because the low sensitivity of smear microscopy makes it is not a good reference standard for calculating these.

All analyses were conducted in SPSS 20 and ReCal [19]. Findings were considered to be statistically significant if *p* < 0.05.

### Ethics

Ethical approval was obtained from the National Bioethics Committee and, since there was no direct contact with patients, the need for informed consent was waived. APOPO’s animal welfare assurance was approved by the Office of Laboratory Animal Welfare.

## Results

### Intra-rater reliability

The median Yule’s Q for the rats was 0.909 (*range 0.73–0.975*; Table 1). Of the 22 animals, 21 demonstrated higher reliability with DOTS-positive than DOTS-negative samples, and one demonstrated no difference between the sample types. Overall, animal results were more reproducible with DOTS-positive samples (Median *=* .978) than DOTS-negative (Median *=* .861), a significant increase in the median of the differences of .077, *p =* .000. Only animals J and V had Yule’s Q values under .8 for all samples. For animal V this was due to lower reproducibility on negative samples (Yule’s Q = .613) because agreement on positive samples was substantial (Yule’s Q = .946).

**Table 1:** Yule’s Q for all, DOTS-positive, and DOTS-negative samples

Rat	All Samples	DOTS-positive Samples	DOTS-negative Samples
A	0.892	0.978	0.844
B	0.958	0.996	0.92
C	0.873	0.997	0.791
D	0.942	0.942	0.904
E	0.94	0.978	0.901
F	0.893	0.979	0.793
G	0.925	0.976	0.878
H	0.942	0.964	0.915
I	0.949	0.981	0.924
J	0.73	0.754	0.754
K	0.955	0.973	0.938
L	0.959	0.997	0.92
M	0.844	0.979	0.762
N	0.95	0.996	0.927
O	0.86	0.972	0.799
P	0.885	0.98	0.79
Q	0.975	0.993	0.962
R	0.852	0.973	0.777
S	0.87	0.877	0.806
T	0.949	0.99	0.92
U	0.879	0.851	0.843
V	0.784	0.946	0.613
Median *(range*)	0.909 (*0.73–0.975*)	0.978 (*0.754–.997*)	0.861 (*0.613–0.962*)

There was a statistically significant, moderate positive correlation between age and Yule’s Q on all samples (*r*_*s*_ (20) = .449, *p* = .036). Mann-Whitney U test was run to determine if there were differences in reproducibility between male and female animals. Distribution of Yule’s Q was similar as assessed by visual inspection. Median Yule’s Q was not significantly different between males (*Median* = 0.94) and females (*Median* = .885), *U* = 69, *z* = .702, *p* = .512.

### Inter-rater reliability

Krippendorff’s alpha for all samples was fair, .344 for group one and .232 for group two, and highest for clinic-positive samples; .437 and .285 for groups one and two respectively (Table 2). Krippendorff’s alpha was lowest for samples designated as negative by the clinics.

**Table 2:** Krippendorff’s alpha and agreement

	Krippendorff’s alpha	Observed agreement	Expected agreement	N Decisions
Group 1				
All	.344	.683	.517	56,155
Positive	.437	.783	.615	8844
Negative	.26	.611	.52	51,612
AFB	.454	.745	.533	1606
1+	.441	.773	.594	3674
2+	.38	.809	.692	3344
3+	.328	.845	.771	220
Group 2				
All	.232	.657	.561	74,404
Positive	.285	.681	.553	9900
Negative	.152	.654	.592	64,504
AFB	.268	.637	.504	1991
1+	.289	.678	.547	4103
2+	.252	.709	.611	3531
3+	.174	.667	.597	275

### Accuracy

Compared to the smear microscopy used in DOTS clinics, rats emitted very few false negatives, resulting in a sensitivity of 93% for group 1 and 94% for group 2 (Table 3).

**Table 3:** Sensitivity of detection rats compared to Ziehl-Neelsen smear microscopy

	TP	FN	Sensitivity *(95% CI)*
Group 1	748	56	93.0 *(91.0–94.7)*
Group 2	845	55	93.9 *(92.0–95.3)*

## Discussion

This study found that the inter-rater reliability of detection rats was fair, which is an important finding given that a common criticism of disease-detection animals is that their performance is variable [9]. Although the performance of the detection rats used in the present study may be variable across another dimension (e.g., time), it was highly consistent across successive presentations of the same sample. This can be construed as a form of test-retest reliability, which is a common measure of one aspect of the quality of a measurement device [20]. Animal J was the only rat to demonstrate poor reliability on both DOTS-negative and DOTS-positive samples. This particular animal required an additional 2 weeks to meet accuracy criteria in early training; additional training did not improve performance. It may be due to an as-yet -unidentified health problem or discrepancies in training procedure. That occasionally individual animals are unreliable is further justification for using groups to asses TB samples.

The inter-rater reliability is classified as “fair” by medical diagnostic standards [18]. This means that there was some variability between the rats in terms of indicating on samples. It must be noted that Krippendorff’s alpha measures observed agreement over the disagreement we would expect by chance. Lower agreement on samples designated as 2+ and 3+ is unsurprising as rats tend to be more accurate over these samples, so even chance arrangement of positive indications would result in high expected agreement. Given that individual rats fail to indicate on some samples that are actually TB-positive, groups of rats are used which substantially increase sensitivity relative to the use of a single animal [6, 9]. The magnitude of the increase depends on the number of rats used and the criterion used (i.e., the number of rats in a group of designated size indicating on the sample) to determine whether or not a sample is considered as rat-positive. The increased sensitivity comes at the cost of decreased specificity [6, 21]. Some rats appear to be “conservative” in their evaluation of samples, in that they have relatively low sensitivity and high specificity relative to culturing, whereas others are more “liberal,” having higher sensitivity but lower specificity. It may be possible to use the performance of individual rats as a basis for configuring groups of rats, and the group criterion for classification of samples as rat-positive, that maximize sensitivity while minimizing specificity, and this is a worthy objective for future research.

The rats’ intra- and inter-rater reliability was significantly higher in DOTS-positive samples than in DOTS-negative samples, which may in part be due to the fact that only correct identifications of DOTS-positive samples were reinforced. There was a statistically significant, but moderate, positive correlation between the age of rats and Yule’s Q. This finding is consistent with the general finding that the performance of animals often becomes less variable as exposure to a given task increases [22, 23]. Interestingly, age may have contributed to the difference in performance in the two groups, which was somewhat better in Group 1 than in Group 2. The median age of rats in Group 1 was 3.8 years, whereas it was 2.4 years for rats in group 2. Moreover the two rats with the lowest Yule’s Q were less than 2.5 years old on 31 August 2015, when data collection ended.

Although the sex of other animals sometimes affects their performance in discrimination tasks similar to those performed by pouched rats in the present study [12, 13], there was no significant difference between male and female animals with respect to intra-rater reliability. The consistently high Yule’s Q values obtained in the present study suggest that both sexes are appropriate for use in operational TB detection task and that variability of performance, at least with respect to test-retest reliability, is not a serious concern when they are so used.

Comparing rat performance to smear microscopy as used in DOTS clinics showed that they are highly sensitive. A previous accuracy study showed that compared to culture, a group of detection rats are 57–72% sensitive and 59–81% specific [24].

## Conclusions

This study showed that the test results of detection rats are consistent within and between animals. Even the lowest performers demonstrate very high agreement when presented with the same sample twice. Agreement among multiple rats is fair, and agreement on smear-positive samples is highest. More experienced rats had higher intra-rater reliability, whereas intra- and inter-rater reliability was independent of animal gender. These results complement previously obtained findings regarding the accuracy of the rats and suggest that the rats produce consistent results in operational settings.

## Additional files

Additional file 1: Group One Intra-Reliability. (XLSX 390 kb)

Additional file 2: Group Two Intra-Reliability. (XLSX 319 kb)

Additional file 3: Group One Inter-Reliability. (XLSX 935 kb)

Additional file 4: Group Two Inter-Reliability. (XLSX 2235 kb)

## Abbreviations

APOPO: Anti-persoonsmijnen ontmijnende product ontwikkeling
DOTS: Directly-observed treatment, short course
FN: False negatives
FP: False positives
IQR: Interquartile range
NPV: Negative predictive value
PPV: Positive predictive value
TB: Tuberculosis
TN: True negatives
TP: True positives
